# Utilizing the transformer mechanism to predict cervical lymph node metastasis in patients with papillary thyroid carcinoma

**DOI:** 10.1371/journal.pone.0345937

**Published:** 2026-04-03

**Authors:** Huiting Chen, Fangqiu Ruan, Li Zhu, Yong Zhuang, Xiaojian Ye, Xinxiu Liu, Jinshu Zeng

**Affiliations:** 1 Department of Ultrasound Imaging, The First Affiliated Hospital, Fujian Medical University, Fuzhou, China; 2 Department of Ultrasound Imaging, National Regional Medical Center, Binhai Campus of the First Affiliated Hospital, Fujian Medical University, Fuzhou, China; 3 Department of Gastrointestinal Surgery 2 Section, Institute of Abdominal Surgery, Key Laboratory of Accurate Diagnosis and Treatment of Cancer, The First Hospital Affiliated to Fujian Medical University, Fuzhou, China; Fondazione Policlinico Universitario Agostino Gemelli IRCCS, ITALY

## Abstract

**Rationale and objective:**

The status of cervical lymph node metastasis(LNM) in Papillary thyroid carcinoma(PTC) can affect the patient’s treatment plan and prognosis. This study aims to develop and validate the application value of Vision transformer (ViT) model in preoperatively predicting cervical LNM in PTC.

**Materials and methods:**

A total of 540 PTC patients were retrospectively reviewed from two hospitals from April 20,2022 to August 20,2023.The ViT model is built based on the two-dimensional rectangular ultrasound image of the primary thyroid tumor, and at the same time, to compare its performance, a deep learning model of the traditional Convolutional neural network (CNN) framework, a ultrasound radiomics combined model(Clinical-Rad model), and clinical model are built.

**Results:**

The ViT model demonstrated an AUC of 0.807 (95% CI: 0.709-0.905) in the internal validation cohort and 0.809 (95% CI: 0.720-0.900) in the external validation cohort. The ViT model’s AUC ranged from 0.807-0.814 across all cohorts, significantly exceeding the clinical model (AUC: 0.595-0.669, P<0.001). While the AUC of the ViT model in the training cohort was slightly lower than that of the combined ultrasound radiomics model (0.814 vs 0.828, P=0.491), it showed significantly higher AUC values in the internal (0.807 vs 0.718, P=0.049) and external validation cohorts (0.809 vs 0.691, P<0.001). Compared to the clinical and combined radiomics models, the ViT model exhibited stable and superior predictive performance for PTC cervical lymph node metastasis.In the internal validation cohort, Doctor C’s net reclassification improvement (NRI) with the ViT model was 0.106 (P=0.022), and the integrated discrimination improvement (IDI) was 0.106 (P=0.023). Doctor D showed NRI and IDI values of 0.113 (P=0.022) and 0.106 (P=0.024), respectively. In the external validation cohort, Doctor C’s NRI and IDI were 0.090 (P=0.024) and 0.106 (P=0.024), while Doctor D had values of 0.011 (P=0.013) and 0.106 (P=0.013). The ViT model enhanced the diagnostic capabilities of both Doctor C, with less clinical experience, and Doctor D, with extensive experience.

**Conclusion:**

The deep learning model based on the Transformer mechanism shows good performance in predicting LNM in PTC patients, which is superior to the clinical model, Clinical-Rad model, and traditional CNN model.

## Introduction

The incidence of thyroid cancer has gradually increased in recent years and has become one of the top ten cancers worldwide in terms of incidence [1]. PTC is the most common pathological subtype of differentiated thyroid cancer [2]. According to the eighth edition of AJCC/UICC TNM staging [3], most PTC patients are in stage I or II, and the 10-year disease-specific survival rate (DSS) can reach 90% [4,5]. Accurate preoperative assessment of LNM is beneficial for formulating appropriate surgical plans [6]. Studies have shown that about 30%−80% of PTC patients develop LNM [7]. In cases where clinical indications are met, patients who are clinically diagnosed as clinically lymph node positive (cN1) commonly undergo therapeutic cervical lymph node dissection [6]. However, due to the complex anatomy of the neck and the problem of incomplete imaging affected by tracheal gas, only about 20%−40% of patients can be diagnosed with regional lymph node metastasis during the clinical ultrasound diagnosis process [8]. In addition, some patients have clinically occult lymph node lesions, and about 80% of postoperative pathology in patients who are clinically lymph node negative (cN0) indicates the presence of occult lymph node metastasis [9,10]. Because the risk of central neck lymph node metastasis is high [11], and lymph node metastasis is related to the risk of disease recurrence and cancer death [12], some scholars suggest that preventive central neck lymph node dissection (pCND) should be performed on cN0 patients, as it helps to accurately stage postoperatively and provide more precise subsequent clinical treatment plans [13–15]. However, no high-level evidence has been found in the current research that performing pCND can reduce the risk of patient recurrence or increase the short-term survival rate, but it may increase the incidence of postoperative complications [16,17]. Therefore, a strict and accurate preoperative assessment of the cervical lymph node status of patients with papillary thyroid carcinoma is needed to optimize the formulation of surgical strategies [18].

Routine neck ultrasound examination is the preferred means of preoperative assessment of neck lymph nodes in PTC patients [19], mainly assessing cervical lymph node metastasis by identifying typical features [20]. However, a retrospective meta-analysis [21] showed that the sensitivity of ultrasound examination for central lymph node metastasis was about 33%; sensitivity and specificity for neck side lymph nodes were about 70% and 88%, respectively, the diagnostic performance is not ideal. In recent years, various methods have been proposed to improve the prediction of thyroid neck lymph node metastasis by ultrasound, including ultrasound angiography, Fine-needle aspiration cytology (FNAC), Fine-needle aspiration-thyroglobulin(FNA-Tg), etc., but their performance and practicability are not satisfactory [22–25]. With the rapid advancement of medical image analysis, non-invasive deep learning approaches based on conventional ultrasound images have emerged as a research focus for predicting cervical lymph node metastasis in PTC. Some studies have utilized classical deep learning models on key frames from 2D ultrasound images to predict lymph node metastasis in thyroid cancer, achieving AUC values of 0.88 ± 0.23 on the training set, 0.88 ± 0.23 on the internal test set, and 0.85 ± 0.24 on an external test cohort, demonstrating good discriminative ability [26]. Another study developed an interpretable multi-model 2D ultrasound-based framework known as the Lateral Lymph Node Metastasis Network (LLNM-Net), which attained an AUC of 0.944 and a predictive performance of 84.7% in a multi-center validation [27]. However,most existing models of this type are still built upon traditional Convolutional Neural Networks(CNN) architectures, which may have limitations in global feature extraction and long-range dependency modelin.

In recent years, with the continuous development of the field of deep learning, deep learning models based on CNN architecture and the well-received Transformer architecture have emerged [28]. ViT model is one that applies the latter to image block sequences, performs well in image classification tasks, does not rely on predefined manual features, and handles images more flexibly and efficiently. This innovation shows the powerful predictive potential of deep learning models, changing the paradigm of computer vision and opening up the possibility of new research methods. Existing research has applied ultrasound image-based ViT models to the detection and diagnosis of thyroid cancer, demonstrating the feasibility of utilizing ViT in ultrasound image analysis studies. Wu et al. developed a natural language processing system for evaluating thyroid ultrasound reports, based on the GatorTron large language model derived from the VIT architecture, which achieved F1 scores exceeding 90% in 14 out of 16 thyroid categories and effectively identified thyroid nodule features [29]. However, current studies on cervical lymph node metastasis in PTC primarily rely on CNN frameworks or traditional radiomics methods [30], failing to fully leverage the advantages of the Transformer architecture in capturing long-range dependencies and subtle metastatic features in ultrasound images. This study aims to explore and validate the application value of the ViT model in PTC cervical lymph node metastasis, and to determine whether it outperforms existing clinical models, radiomics-integrated models, and traditional CNN models, a gap for which sufficient evidence and research support are currently lacking.

Therefore, this study aims to build a prediction model for LNM based on the Transformer mechanism, and compare it with clinical models, radiomic models, and traditional CNN models, in order to find the optimal prediction model. This will realize the precise prediction of preoperative thyroid papillary carcinoma patient neck lymph node metastasis, providing more diagnostic information to support the formulation of clinical treatment strategies.

## Materials and methods

### Study population

This retrospective study, which has been approved by The First Hospital Affiliated to Fujian Medical University ethics committee (MTCA, ECFAH of FMU[2015]084−2), and has been exempt from the requirement for informed consent, collected 483 cases from April 20,2022 to August 20,2023 in the First Hospital, and 111 cases from February 20,2023 to August 20,2023 in the Second Hospital, all confirmed by postoperative pathology as PTC.The study complied with the ethical standards outlined in the Declaration of Helsinki.Patients were divided into cervical lymph node metastasis group (LNM+) and non-metastasis group (LNM-) according to postoperative pathological results. Inclusion criteria: (1) underwent total thyroid lobectomy or unilateral thyroid lobectomy; (2) at least underwent ipsilateral central area lymph node dissection; (3) All confirmed as PTC by postoperative pathology; (4) preoperative thyroid and cervical lymph node ultrasound examination were conducted. Exclusion criteria: (1) incomplete clinical and postoperative pathological data; (2) poor ultrasound image quality (such as low image resolution, obvious noise, etc.); (3) previously accepted thyroid or neck surgery, such as radiofrequency ablation treatment; (4) distant metastasis; (5) history of other malignant tumors. Both hospitals have the same inclusion and exclusion criteria.

### Collection of ultrasound image features of thyroid primary tumors

This study utilized ultrasound imaging equipment including PHILIPS (EPIQ 5), GE (Voluson E10), Mindray (Resona 7S, Resona R9G), and SIEMENS (ACUSON Sequoia), with linear array transducers (5–15MHz) for image acquisition. Preoperative ultrasound images of primary thyroid tumors were retrospectively collected from patients who met the inclusion and exclusion criteria, selecting the most representative ultrasound imaging data. A total of 540 two-dimensional ultrasound images of primary thyroid tumors from 540 patients were gathered. Based on these thyroid ultrasound images, two ultrasound physicians (Doctor A and Doctor B), both with 10 years of neck diagnosis experience, jointly assessed the sonographic characteristics of the primary thyroid tumors. When discrepancies arose in their interpretations of image characteristics, the final decision was made by another ultrasound physician with 15 years of thyroid diagnosis experience. During the course of the study, all doctors were not allowed to access patients’ clinical and histopathological information.

**Fig 1:**
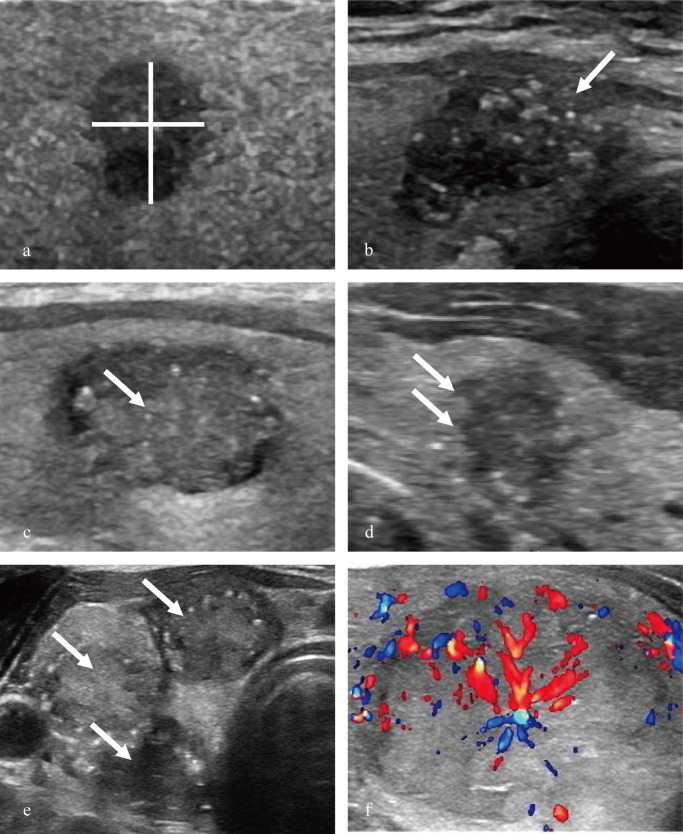
Papillary thyroid carcinoma two-dimensional ultrasound image a-e and color Doppler flow image f. **a** The aspect ratio of the nodule is taller than high. **b** The nodule is irregular in shape and has an unclear boundary with the thyroid capsule (arrows). **c** Microcalcification is seen within the nodule (arrows). **d** Burrs and horns are seen on the edge of the nodule (arrows). **e** Multiple nodules are found and fused into a mass (arrows). **f** Rich blood flow signals are seen within the nodule (arrows).

This study, according to the ACR TI-RADS (American College of Radiology; Thyroid Imaging Reporting and Data System) [20], includes the thyroid nodule features for evaluation as seen in the Fig 1 shows some PTC ultrasound image features(S2 File).

### Assessment of cervical lymph node metastasis status

**Fig 2:**
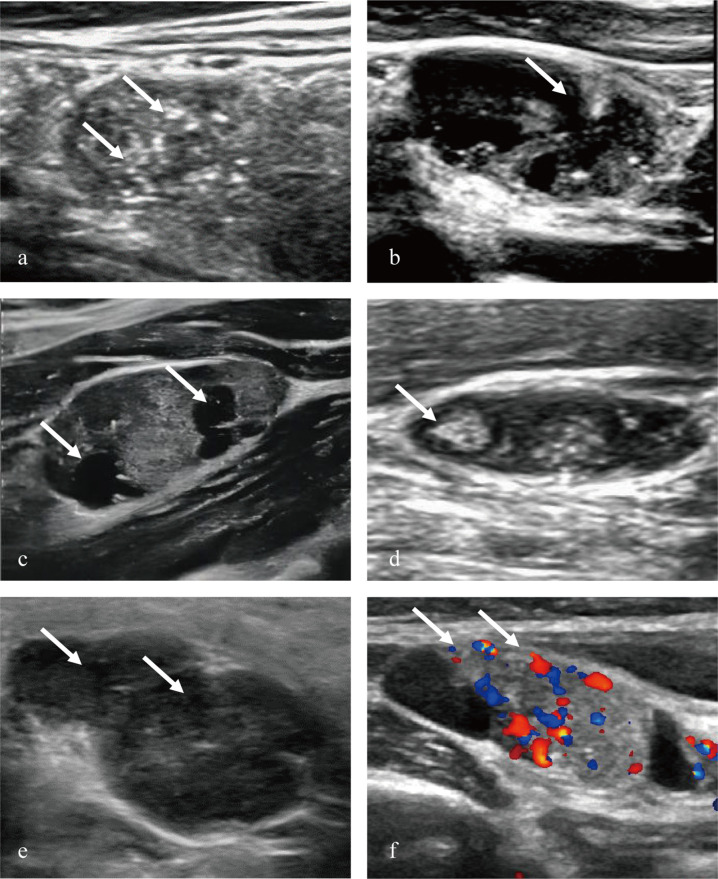
Two-dimensional ultrasound images a-e and color Doppler flow images f of cervical lymph node metastasis in papillary thyroid carcinoma. **a** Microcalcification was found in lymph node (arrows).**b** lymphatic portal misalignment in lymph node (arrows), and the boundary between cortex and medulla was unclear (arrows). **c** partial cystic degeneration was found in lymph node (arrows).**d** hyperechoic mass was found in lymph node (arrows).**e** multiple lymph node fusions were found in lymph node (arrows).**f** blood flow signal was found at the edge of lymph node (arrows).

Two ultrasound physicians (C and D, with 5 and 15 years of thyroid diagnostic experience, respectively) independently reviewed the patients’ cervical lymph node ultrasound images. A lymph node was considered metastatic if it demonstrated any one sonographic feature of microcalcification or cystic change within the lymph node, or presented two or more other lymph node metastasis features(such as a round or near-round shape of lymph node, loss of hilum structure, presence of a hyperechoic mass within the lymph node, rich or relatively rich blood supply, or peripheral or mixed blood flow distribution, details in S2 File). The results of the two physicians’ diagnoses were recorded, with neither physician having access to the patients’ histopathological results throughout the process. Fig 2 shows some ultrasound features of suspected metastatic lymph nodes in PTC.

### Clinical model construction

**Fig 3:**
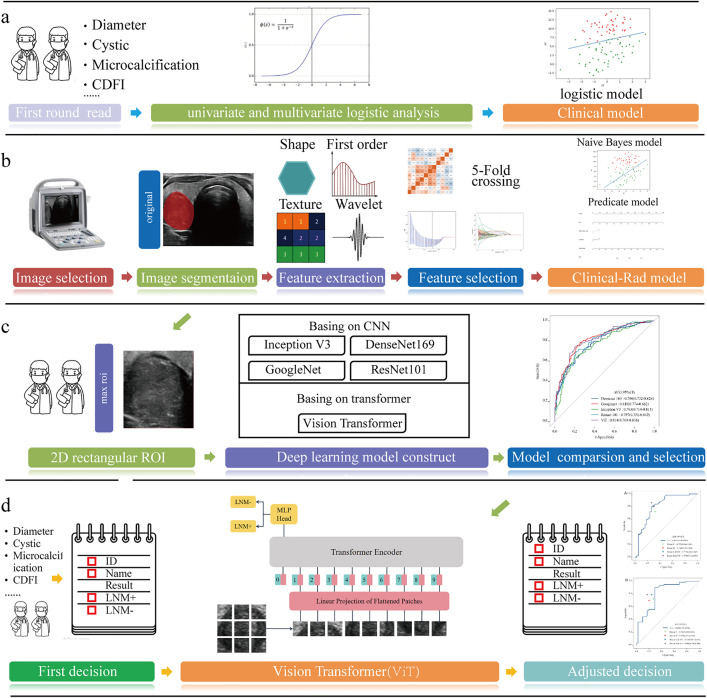
Research flowcharts. **a** Clinical model establishment process. **b** Clinical-Rad model establishment process. **c** deep learning model establishment process and ViT principle diagram. **d** model-assisted ultrasound doctor diagnostic process.

The clinical model research process is shown in Fig 3a. The clinical features and ultrasound image features of the patients included in the training cohort were analyzed using univariate and multivariate logistic regression to determine clinical independent predictors and construct Clinical model. Relevant statistics were calculated to assess the diagnostic performance of the prediction model

### Ultrasonic image processing and labeling

This study implemented systematic image preprocessing using SimpleITK to eliminate the effects of image quality and technical variations, which included: Normalization to unify brightness and contrast; Resampling to standardize pixel spacing; Bin width adjustment for consistent gray-scale distribution; Filtering operations to enhance image quality. The SitkBSpline interpolation method was used to improve the accuracy and comparability of image feature extraction, and these images were resampled to a voxel spacing of 1*1mm3.

An experienced physician A manually outlined the Region of Interest (ROI) on the preprocessed ultrasound images twice using 3D slicer (version 5.2.2), with a one-month interval between the two sessions, to obtain the mask of the primary thyroid lesion. Before outlining, the irrelevant areas of the original ultrasound image were clipped (mainly the dark areas near the image periphery and body markers). Another experienced physician B randomly selected ultrasound images from 30 patients in the training cohort and outlined the ROI following the same steps. Both doctors did not have access to patients’ histopathological results before and after participating in the research. The Intra-class correlation (ICC) was then calculated to assess the stability and repeatability of the sonographic radiomics. An ICC > 0.75 indicates good intra and inter-group consistency.

### Radiomics feature extraction

This study used the Pyradiomics (version 3.0.1, based on Python) to extract radiomic features. A total of 464 radiomic features were extracted from each ultrasound original image and images after wavelet transformation, including 9 shape features, 18 first-order features, 73 second-order and higher texture features, and 364 features after wavelet transformation. Detailed parameters and configuration files for radiomic feature extraction are described in the **supplementary materials** S1 File and on the **PyRadiomics website** (https://pyradiomics.readthedocs.io/en/latest).

### Radiomics feature selection and model development

The research process for Clinical-Rad model is shown in Fig 3b. The specific process is as follows: (1) Standardize (Z-score Normalization) the radiomics feature data. (2) Use independent sample T-tests to eliminate irrelevant features with *P* > 0.05, use Pearson correlation tests to calculate the correlation between features, and eliminate features with a Pearson correlation coefficient≥0.9. (3) Use the Least Absolute Shrinkage and Selection Operator (LASSO) method to select features based on 5-fold cross validation. (4) Based on the LASSO results, we use an Naive Bayes (NB) model, with the training process using an internal validation cohort to optimize the model parameters. The predicted probability of LNM for each PTC patient is calculated by linearly combining each selected feature with its regularization coefficient, acting as a radiomics score (RadScore). (5) The RadScore and independent clinical predictors are combined to establish the ultrasound radiomics combined model(Clinical-Rad model).

### Deep learning model development

The deep learning model research process is shown in Fig 3c. Based on the two-dimensional ultrasound image mask of primary thyroid tumors, a two-dimensional rectangular ROI containing the largest tumor area is cut from each ultrasound image. The division method and dataset of these images are the same as the establishment and verification process of the radiomics model. A total of five deep learning models have been established to study this data, including ViT models based on self-attention mechanisms (Fig 3c), and traditional CNN models based on convolutional neural networks, including Inception V3, DenseNet 169, GoogLeNet, and ResNet101 (pretrained on the ImageNet dataset). Transfer learning based on ImageNet has been used in many medical studies. We use a global fine-tuning strategy to update the parameters to make the model applicable to the prediction of PTC cervical lymph node metastasis. The model evaluation metrics are the same as the process of establishing the radiomics model. The model development consisted of two phases based on a fixed 8:2 split of the dataset (N = 438) into a training set (n = 350) and a held-out validation set (n = 88) via stratified random sampling. For hyperparameter tuning on the training set, a 10-fold cross-validation was employed: the training set was partitioned into 10 folds; in each iteration, the model was trained on 9 folds and validated on the remaining fold, rotating until all folds served as validation. The hyperparameters with the best average performance across the 10 folds were selected.The model was then retrained on the entire training set (n = 350) and evaluated on the untouched validation set (n = 88) to obtain the final unbiased performance metrics. The training process of all deep learning models includes forward propagation, backpropagation, gradient descent, and coefficient updates. In the training phase, the two-dimensional rectangular ROI is inputted into the network. The prediction result is obtained through forward propagation. The loss is calculated with cross-entropy as the loss function, and then the gradient of the loss function is calculated through backpropagation. The network parameters are adjusted by gradient descent to minimize the loss function, and the output of the network is finally used as the classification result. However, what is different from the traditional CNN model is that ViT will first divide the inputted two-dimensional rectangular ROI into fixed-size patches and convert them into embedding vectors, encode and predict categories through the Transformer mechanism, and then calculate the loss based on the prediction results and real labels, perform backpropagation and parameter updates until the training is over.

To ensure reproducibility, the complete experimental setup is detailed as follows: During the training process, all images inputted into the model are adjusted to 224*224 pixels and are normalized. To alleviate overfitting and the impact of sample imbalance, data augmentation techniques including random horizontal flipping and random cropping were applied. The training hyperparameters were consistent across all models: Stochastic Gradient Descent (SGD) was used as the optimizer with a momentum of 0.9, an initial learning rate of 0.01, a batch size of 32, and training was conducted for 80 epochs. A learning rate scheduler was implemented, reducing the learning rate by a factor of 10 if the validation accuracy did not improve for 10 consecutive epochs. The model checkpoint with the smallest loss on the validation set was selected for final evaluation. All models were implemented using PyTorch 1.13.1. The ViT architecture was optimized via the aforementioned 10-fold cross-validation. The final selected hyperparameters for the ViT model were: patch size of 32, embedding dimension (dim) of 1024, Transformer depth of 6, attention heads of 16, and MLP dimension of 768. All experiments were conducted on a computer system equipped with 64 GB of system RAM and an NVIDIA GeForce RTX 4060 GPU (24 GB VRAM, CUDA 12.1). The operating system was Ubuntu 22.04. Code execution utilized Python 3.10.9 with key libraries including NumPy 1.24.3, pandas 2.0.0, PyTorch 1.13.1, and scikit-learn 1.2.2. The detailed derivation of time estimates and runtime memory usage is provided in S2 Table.

### Statistical analysis

Statistical analyses and graphical production were performed using Python (version 3.10.9) and R-software (version 4.3.2, http://www.R-project.org). Clinical characteristics of patients were represented by frequency (percentage) for categorical variables and mean (± standard deviation) or median (interquartile range) for continuous variables. Continuous variables were evaluated using independent samples T-test or the Mann-Whitney U test, while categorical variables were assessed using the Chi-square test or Fisher’s exact probability method. The AUC value and its 95% CI were used to compare the discrimination between different models, and Delong’s test was used to compare differences in AUC values between models. The NRI and IDI were used to assess the classification accuracy between different models. Other performance metrics used to evaluate the models included accuracy, sensitivity, specificity, negative Predictive Value, and Positive Predictive Value. All testing methods used in this study were two-sided tests, with *P* < 0.05 considered as statistically significant.

## Result

### Patient characteristics

**Fig 4:**
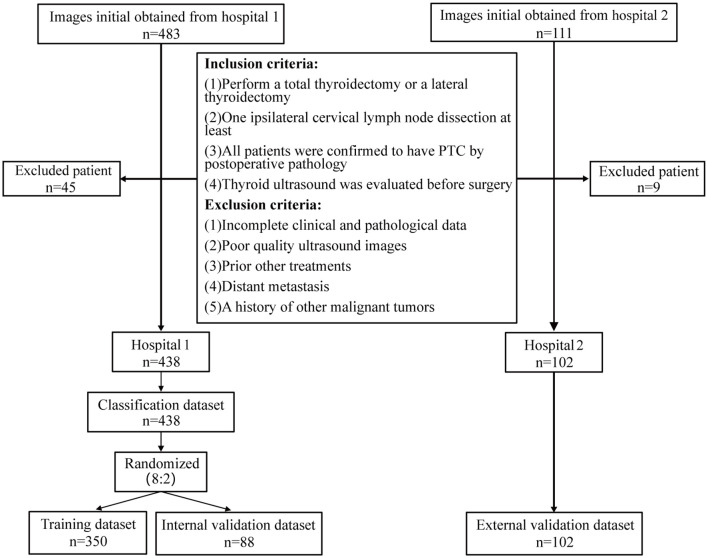
Conclusion and exclusion criteria for patients.

After screening, a total of 438 PTC patients from the first hospital were included, which were randomly divided into training and internal validation sets at an 8:2 ratio. There were 350 cases in the training set, including 178 with LNM+ and 172 with LNM-. The internal validation set included 88 cases, with 50 being LNM+ and 38 being LNM-. A total of 102 PTC patients were included from the second hospital, serving as an external validation set, with 41 being LNM+ and 61 being LNM- (Fig 4). The rates of cervical lymph node metastasis in the training set (n = 350), internal validation set (n = 88), and external validation set (n = 102) were 48.6%, 43.2%, and 40.2%, respectively. When comparing the three research sets, there were statistically significant differences in FT3, TPOAb, TGAb, etc. (*P* < 0.05), while other feature differences were not statistically significant (*P* > 0.05). The baseline information in the three sets was basically balanced (S1 Table).

### Performance analysis of clinical model

A univariate logistic analysis of the clinical data and ultrasound features of PTC patients indicates that tumor maximum diameter (*P* < 0.001), length-width ratio (*P* < 0.001), microcalcification (*P* = 0.015), and internal blood flow signal (*P* = 0.001) are associated with PTC cervical lymph node metastasis (S1a Fig). Further multivariate logistic analysis showed that tumor maximum diameter (≥1 cm) (OR=1.270, 95%CI: 1.151–1.402, *P* < 0.001) and length-width ratio (>1) (OR=0.880, 95%CI: 0.802–0.966, *P* = 0.024) are independent predictive factors for PTC cervical lymph node metastasis (S1b Fig).

Based on the results of the multivariate logistic analysis, clinical model was constructed incorporating the variables tumor maximum diameter and length-width ratio. The AUC of the clinical model in the training set, internal validation set, and external validation set were 0.669 (95%CI: 0.585–0.798), 0.606 (95%CI: 0.496–0.715), and 0.595 (95%CI: 0.491–0.699) respectively. (Table 1).

**Table 1:** Performance Comparison of Clinical Model, Clinical-Rad Model, and ViT.

Model and Cohort	AUC	Accuracy	Sensitivity	Specificity	PPV	NPV	*P* value^a^
Training validation							
Clinical model	0.669	0.640	0.482	0.802	0.717	0.600	<0.001
Clinical-Rad model	0.828	0.786	0.787	0.785	0.791	0.780	0.491
ViT	0.814	0.766	0.730	0.802	0.793	0.742	Reference
Internal validation							
Clinical model	0.606	0.602	0.460	0.789	0.742	0.528	<0.001
Clinical-Rad model	0.718	0.727	0.760	0.684	0.760	0.684	0.049
ViT	0.807	0.773	0.800	0.737	0.800	0.737	Reference
External validation							
Clinical model	0.595	0.588	0.443	0.805	0.771	0.493	<0.001
Clinical-Rad model	0.691	0.706	0.689	0.732	0.792	0.612	<0.001
ViT	0.809	0.824	0.902	0.707	0.821	0.829	Reference

**Abbreviations:** ViT,Vision transformer; AUC, Area under the curve; PPV, Positive predictive value; NPV, Negative predictive value.^a^P value was calculated by the Delong test.

### Performance analysis of radiomic model

The extracted radiomics features were filtered. A total of 11 features were finally included to calculate the Radscore. Specific radiomics features are shown in S2 Fig. By combining the Radscore with independent clinical predictors of LNM (tumor maximum diameter, length-width ratio), a radiomics combined model(Clinical-Rad model) was constructed. The correlation analysis between clinical independent predictors and radiomics features, along with the correlation analysis among radiomics features, is shown in S3 Fig.

The evaluation metrics of Clinical-Rad model are detailed in Table 1. The model performed well, with AUC values in the training set, internal validation set and external validation set of 0.828 (95%CI: 0.785–0.871), 0.718 (95%CI: 0.609–0.828), and 0.691 (95%CI: 0.585–0.798) respectively.

### Performance analysis of deep learning model

The specific metrics of the established deep learning models are detailed in Table 2. In the training set, the deep learning models all demonstrated good predictive efficiency for cervical lymph node metastasis in PTC patients, with the highest AUC values being the ViT model and the GoogLeNet model, at 0.814 (95%CI: 0.769–0.858) and 0.818 (95%CI: 0.774–0.862), respectively. In the internal validation set, the ViT model had an AUC value of 0.807 (95%CI: 0.709–0.905) (ViT vs GoogLeNet, *P* = 0.017). In the external validation queue, the AUC was 0.809 (95%CI: 0.720–0.900) (ViT vs GoogLeNet, *P* = 0.190). Comprehensive comparison shows that the overall performance of the ViT model is the best.

**Table 2:** The performance comparison of different deep learning models.

Model and Cohort	AUC	Accuracy	Sensitivity	Specificity	PPV	NPV	*P* value^a^
Training validation							
ViT	0.814	0.766	0.730	0.802	0.793	0.742	Reference
Densenet 169	0.780	0.703	0.612	0.797	0.757	0.612	0.160
Googlenet	0.818	0.766	0.753	0.779	0.779	0.753	0.843
Inception V3	0.763	0.709	0.624	0.797	0.760	0.672	0.079
Resnet 101	0.797	0.737	0.713	0.762	0.756	0.720	0.513
Internal validation							
ViT	0.807	0.773	0.800	0.737	0.800	0.737	Reference
Densenet 169	0.750	0.739	0.840	0.605	0.734	0.742	0.071
Googlenet	0.768	0.761	0.820	0.684	0.774	0.743	0.017
Inception V3	0.771	0.761	0.880	0.605	0.746	0.793	0.178
Resnet 101	0.772	0.761	0.840	0.658	0.764	0.758	0.300
External validation							
ViT	0.809	0.824	0.902	0.707	0.821	0.829	Reference
Densenet 169	0.767	0.745	0.803	0.659	0.778	0.692	0.258
Googlenet	0.767	0.716	0.721	0.707	0.786	0.630	0.190
Inception V3	0.761	0.706	0.623	0.829	0.844	0.596	0.153
Resnet 101	0.781	0.696	0.639	0.780	0.812	0.593	0.467

**Abbreviations:** ViT, Vision transformer; AUC, Area under the curve; PPV, Positive predictive value; NPV, Negative predictive value.^a^P value was calculated by the Delong test.

### Comprehensive analysis of clinical model, Clinical-Rad model and ViT model

**Fig 5:**
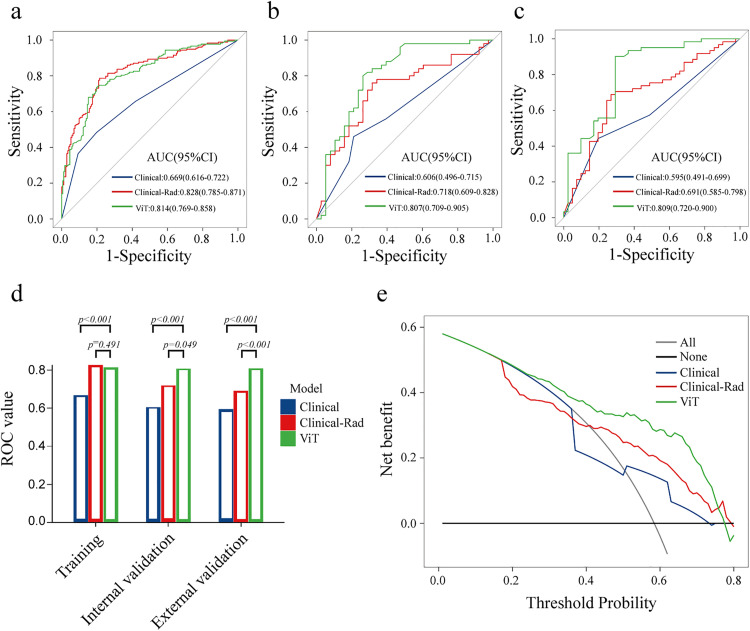
The AUC of the ViT model, clinical model and combined radiomics model. **a**, **b** and **c** respectively refer to the training set, internal validation queue and external validation queue. **d** Delong test results of the ViT model, clinical model and combined radiomics model. **e** Decision curve analysis of the ViT model, Clinical model and Clinical-Rad model.

The performance metrics of the prediction models in each set are detailed in Table 1. In the training set, the AUC values of the ViT model, clinical model and Clinical-Rad model were 0.814 (95%CI: 0.769–0.858), 0.669 (95%CI: 0.614–0.723) and 0.828 (95%CI: 0.785–0.871) respectively (ViT vs Clinical model, *P* < 0.001; ViT vs Clinical-Rad model, *P* = 0.491). In the internal validation set, the AUC of the ViT model was 0.807 (95%CI: 0.709–0.905), which was better than Clinical-Rad model (AUC: 0.718, 95%CI: 0.609–0.828, *P* = 0.049) and clinical model (AUC: 0.606, 95%CI: 0.496–0.715, *P* < 0.001). In the external validation set, the ViT model, with an AUC of 0.809 (95%CI: 0.720–0.900), also showed better predictive performance than both Clinical-Rad model and clinical model (*P* < 0.001). The ROC curves of the three models are shown in Fig 5a–d. Overall, the ViT model had the best comprehensive ability. The clinical utility of the predictive models was further evaluated using the Decision Curve Analysis (DCA) (Fig 5e). The results showed that the threshold range for the net benefit of the ViT model was larger than the other models, and the curve of the ViT model was above the others in most ranges, meaning that the ViT model could achieve better clinical net benefits in most threshold ranges.

### Diagnostic performance of the sonographers with or without a ViT model

**Fig 6:**
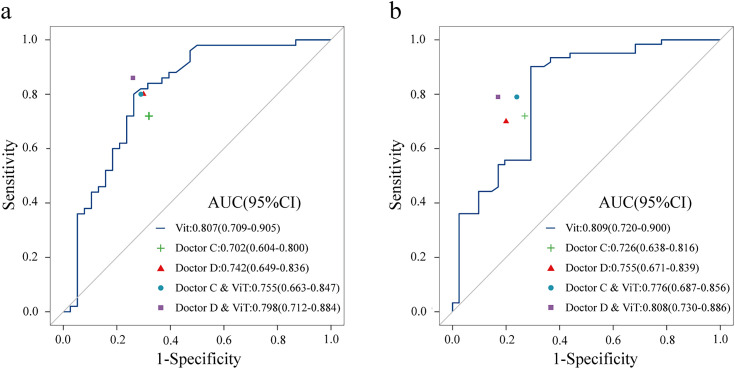
Performance comparison between radiologists with and without ViT assistance in the internal testing cohort(a) and external-testing cohort(b). **Doctor C** junior experience. **Doctor D** senior experience.

The diagnostic performance of ultrasound physicians diagnosing PTC cervical lymph node metastasis with and without the assistance of the ViT model is detailed in Table 3 and Fig 6. Ultrasound doctor C’s overall performance improved with the assistance of the ViT model. In the internal validation set, doctor C’s AUC value increased from 0.702 (95%CI: 0.604–0.800) to 0.755 (95%CI: 0.663–0.847) (*P* = 0.023), the accuracy improved from 70.5% to 76.1%, the sensitivity increased from 72.0% to 80.0%, the specificity improved from 68.4% to 71.1%, the PPV improved from 75.0% to 78.4%, and the NPV improved from 65.0% to 73.0%. A similar trend was observed in the external validation set. In the internal and external validation sets, the AUC values of the relatively experienced doctor D increased by 0.056 (*P* = 0.024) and 0.053 (*P* = 0.013), respectively, accuracy increased by 5.70% and 5.90%, sensitivity increased by 6.00% and 8.20%, specificity increased by 5.30% and 2.40%, PPV increased by 4.20% and 3.00%, and NPV increased by 7.80% and 7.60%.

**Table 3:** Compare the diagnostic performance between ultrasound doctors with and without the assistance of the ViT model.

Model and Cohort	AUC	Accuracy	Sensitivity	Specificity	PPV	NPV	*P* value^a^
internal validation							
Doctor C	0.702	0.705	0.720	0.684	0.750	0.650	Reference
Doctor C and ViT	0.755	0.761	0.800	0.711	0.784	0.730	0.023
internal validation							
Doctor D	0.742	0.750	0.800	0.684	0.769	0.722	Reference
Doctor D and ViT	0.798	0.807	0.860	0.737	0.811	0.800	0.024
external validation							
Doctor C	0.726	0.725	0.721	0.732	0.800	0.638	Reference
Doctor C and ViT	0.776	0.775	0.787	0.756	0.828	0.705	0.025
external validation							
Doctor D	0.755	0.745	0.705	0.805	0.843	0.647	Reference
Doctor D and ViT	0.808	0.804	0.787	0.829	0.873	0.723	0.013

**Abbreviations:** ViT,Vision transformer; AUC, Area under the curve; PPV, Positive predictive value; NPV, Negative predictive value..^a^P value was calculated by the Delong test.

In the internal validation set, compared to the independent diagnosis by doctor C, the NRI and IDI with the assistance of the ViT model were 0.106 (*P* = 0.022) and 0.106 (*P* = 0.023) respectively, and for doctor D, the NRI and IDI were 0.113 (*P* = 0.022) and 0.106 (*P* = 0.024) respectively. In the external validation set, the NRI and IDI for doctor C were 0.090 (*P* = 0.024) and 0.106 (*P* = 0.024) respectively, and for doctor D, they were 0.011 (*P* = 0.013) and 0.106 (*P* = 0.013) respectively. This suggests that the assistance of the ViT model can help clinical doctors improve the diagnostic accuracy for LNM.

## Discussion

The status of cervical lymph node metastasis in PTC has an impact on the patient’s diagnosis, treatment plan, and prognosis. Therefore, it’s crucial to improve the detection of preoperative cervical lymph node metastasis in PTC patients. In this study, we constructed and validated a ViT model based on the Transformer mechanism to predict the cervical lymph node metastasis status in PTC. The results showed that the AUC of the training queue was 0.814 (95% CI: 0.769–0.858), the internal validation queue AUC was 0.807 (95% CI: 0.709–0.905), and the external validation queue AUC was 0.809 (95% CI: 0.720–0.900). The model showed good predictive performance in all queues, suggesting that the ViT model is a potentially feasible method for predicting cervical lymph node metastasis in PTC. It can more accurately predict the invasiveness of the tumor preoperatively, thereby making more individualized and precise clinical decisions.

Currently, conventional ultrasound examinations have become one of the most commonly used preoperative imaging procedures due to their convenience and safety. However, based on typical LNM characteristics, satisfactory sensitivity and specificity have not yet been achieved [31,32]. This is in line with the results of the assessments of LNM by the two clinical doctors in this study. Ultrasound examinations are easily influenced by the subjective judgment of doctors and largely depend on the professional experience and knowledge of ultrasound physicians. In this study, doctors with different work experiences also showed differences in the judgment of LNM. Whether in the internal validation set or in the external validation set, doctors with rich work experience had better comprehensive judgment ability for LNM than those with relatively less work experience.

Some studies have found that PTC neck lymph node metastasis is closely related to patient age, multifocal tumor, tumor size, BRAF V600E gene, and other factors, and a clinical model has been established based on this [33–35]. The results of univariate and multivariate analyses in this study show that the largest tumor diameter (≥1 cm) (OR: 1.270, 95%CI: 1.151–1.401), and the ratio of length to width (>1) (OR: 0.880, 95%CI: 0.802–0.966) are independent predictive factors influencing PTC neck lymph node metastasis. In most studies, tumor size is also considered an important predictive factor for PTC patient LNM [36]. Researchers [37] believe that small thyroid tumors with the largest diameter <1 cm often show a “taller than wide” morphology (indicating a length to width ratio > 1), but as the tumor grows, the tumor morphology tends to be “wider than tall” (indicating a length to width ratio < 1). Tumors larger than 1 cm tend to grow along the horizontal axis, which can change the shape of the tumor from “taller than wide” to “wider than tall”. The long axis of the tumor is parallel to the thyroid capsule, and the inner layer of the thyroid capsule can form multiple fiber bundles that penetrate into the gland substance. These bundles contain a large number of blood vessels and lymphatic vessels, which may increase the possibility of lymph node metastasis [38]. At the same time, as the tumor grows, the number of new blood vessels inside it rapidly increases, and the active blood vessels inside the tumor may also increase the risk of lymph node metastasis [39]. This is consistent with the results of this study that the largest tumor diameter (≥1 cm) is a risk factor, and the length-width ratio (>1) is a protective factor.

However, the independent predictive factors included in the model in different studies vary, showing some heterogeneity [33–35]. The results of the model established by clinical and ultrasound characteristics show that its overall performance in predicting PTC cervical lymph node metastasis is not good [40,41]. The AUC value of the clinical model established in this study is only 0.669 (95%CI: 0.585–0.798), and the accuracy, sensitivity, and specificity are also not satisfactory. Considering the wide application of ultrasound in the diagnosis of thyroid malignancies and cervical lymph node metastasis, and its lower cost, convenience, and no radiation-risk compared to other images and invasive methods (like enhanced CT, cervical lymph node biopsy, etc.), this study attempts to extract more information from the ultrasound images of thyroid primary tumors to create a non-invasive, low-burden predictive method.

Some studies have reported that models built based on ultrasonographic radiomics combined with clinical features show good performance in predicting PTC cervical lymph node metastasis, with AUC values reaching approximately 0.75–0.85 [42–44]. Based on these studies, this study has combined clinical independent predictive factors and radiomics scores to establish an ultrasound radiomics combined model. The model performs relatively well in the training queue, with an AUC value of 0.828 (95%CI: 0.785–0.871). However, in the internal and external validation queues, its predictive performance is not as expected, with the AUC value being only 0.718 (95%CI: 0.609–0.828) and 0.691 (95%CI: 0.585–0.798). This is slightly different from the results of previous studies, possibly due to heterogeneity in study methods, subjects, and inspection techniques. However, such heterogeneity to some extent can reflect the real, complex, and diverse clinical environment, suggesting that the model may face certain difficulties in dealing with the real clinical environment.

In comparison, deep learning methods use the original pixels of the input image, mine and quantify microscopic imaging features in medical image data, and use multiple convolutional and fully connected layers to learn complex features to achieve classification and prediction tasks [45,46]. Some research has been based on deep learning algorithms [47,48], using ultrasound images to construct predictive models, showing excellent performance in different queues, and superior to ultrasound radiomics models. This is consistent with the performance of the five deep learning models established in this study. This consistency to a certain extent shows that it might be more appropriate to use deep learning network frameworks to solve certain specific tasks in a complex and changing clinical environment than to use machine learning models. In this study, compared with other deep learning models, the combined ultrasonic radiomics model, and the clinical model, the ViT model showed better predictive performance and generalization ability in internal and external validation queues. The performance differences between different CNN models may be attributed to the differences in the internal architecture of the network [49]. It can handle global information from the beginning and process multiple image blocks in parallel, making it more efficient at handling classification tasks. On the other hand, traditional CNNs need to go through multiple convolutions to achieve similar effects, and due to the local connection characteristics of the convolution layer, parallel processing cannot be fully achieved.

This superior performance, however, comes with computational considerations. Our efficiency analysis revealed that the ViT model required approximately 1.8 times longer to train than a ResNet101 benchmark. More critically for clinical deployment, its inference latency per image was also 1.8 times slower. Nevertheless, with a processing capability of approximately 60 frames per second, the ViT model still operates well within real-time constraints for ultrasound analysis. This presents a meaningful accuracy-efficiency trade-off. In settings where diagnostic accuracy is paramount and computational resources are adequate, the performance advantage of ViT justifies its cost. Conversely, in resource-constrained environments prioritizing inference speed, conventional CNNs may offer a more balanced solution. Future work should aim to bridge this efficiency gap through lightweight ViT variants.

Furthermore, unlike previous studies that only evaluated the model’s predictive performance [42], this study also emphasized the complementary roles between ultrasound clinicians and prediction models, effectively combining qualitative and quantitative imaging evaluations. The study shows that both clinicians, with varying levels of experience, improved their diagnostic accuracy in both the internal and external validation queues, with statistically significant differences. With the help of the ViT model, clinicians with relatively less clinical experience can achieve a diagnostic level similar to those with more experience. This suggests the reliability of the ViT model as an auxiliary diagnostic tool, which can effectively reduce misdiagnosis or missed diagnosis due to lack of clinical experience, providing more accurate diagnostic information for the clinic.

Although the proposed ViT model demonstrates encouraging performance, several limitations of this study warrant careful consideration to guide future research and clinical translation. First, this retrospective study may be subject to selection bias, as patients with incomplete data were excluded. While the inclusion of an external validation cohort from a second hospital enhances reliability, both participating centers are from a similar healthcare context. This limits our assessment of the model’s generalizability to unseen institutions with potentially different patient demographics, clinical practices, or ultrasound equipment ecosystems. Future prospective, multi-center studies involving more diverse populations are essential to rigorously validate the model’s robustness and mitigate this potential sampling bias.Second, the “black-box” nature of the Vision Transformer architecture presents a significant interpretability challenge for clinical adoption. Although ViT’s self-attention mechanism can, in principle, highlight informative image regions, providing human-intelligible explanations for individual predictions remains non-trivial. This inherent lack of transparency may hinder clinical trust. Future work should prioritize integrating explainable AI techniques (e.g., attention rollout, gradient-based saliency maps) specifically tailored for medical ViT to bridge this gap and provide clinicians with actionable insights.Third, the variability in ultrasound machine brands and acquisition parameters across patients, while reflective of real-world conditions, introduces technical heterogeneity. Despite our standardization efforts in preprocessing, this variability may act as a confounding factor, influencing the model’s stability and the reproducibility of our results. Developing and adhering to a unified imaging protocol in future prospective studies is crucial to minimize this technical variance.Finally, the observed class distribution imbalance across datasets, though mitigated via a weighted loss function and data augmentation, could still affect model calibration and performance estimation, particularly for the minority class. More advanced techniques for handling imbalance should be explored in subsequent work.

## Conclusion

The preoperative detection of cervical lymph node metastasis in PTC patients is crucial for the choice of treatment plan and disease prognosis. The ViT model established in this study based on the Transformer mechanism can relatively well predict cervical lymph node metastasis through the ultrasound images of primary PTC tumors. It demonstrates better predictive performance than other CNN models, combined ultrasound radiomics models, and clinical models, and is expected to provide a reliable basis for clinicians to develop individualized treatment plans. However, the predictive potential and application value of the ViT shown in this study still need to be further verified by prospective studies.

## Supporting information

S1 Fig The forest plot of univariate and multivariate Logistic regression analysis. a and b represent the results of univariate and multivariate analysis.
(TIF)

S2 Fig Graph of 11 selected radiomics features and their weight coefficients. (TIF)

S3 Fig Correlation analysis between clinical predictors and radiomics features (a); Correlation analysis between radiomics features (b). (TIF)

S1 Table Clinicopathological and sonographic characteristics of patients in PTC by cervical lymph node status. (DOCX)

S2 Table Computational complexity comparison of deep learning models. (XLSX)

S1 File Parameters and configuration files for radiomic feature extraction. (DOCX)

S2 File Ultrasound features of thyroid nodules and lymph node. (DOCX)
