# Supplementary figures and images for: Utilizing the transformer mechanism to predict cervical lymph node metastasis in patients with papillary thyroid carcinoma
Source: PLoS One. 2026 Apr 3;21(4):e0345937. doi: 10.1371/journal.pone.0345937 (PMC13048401; doi:10.1371/journal.pone.0345937)

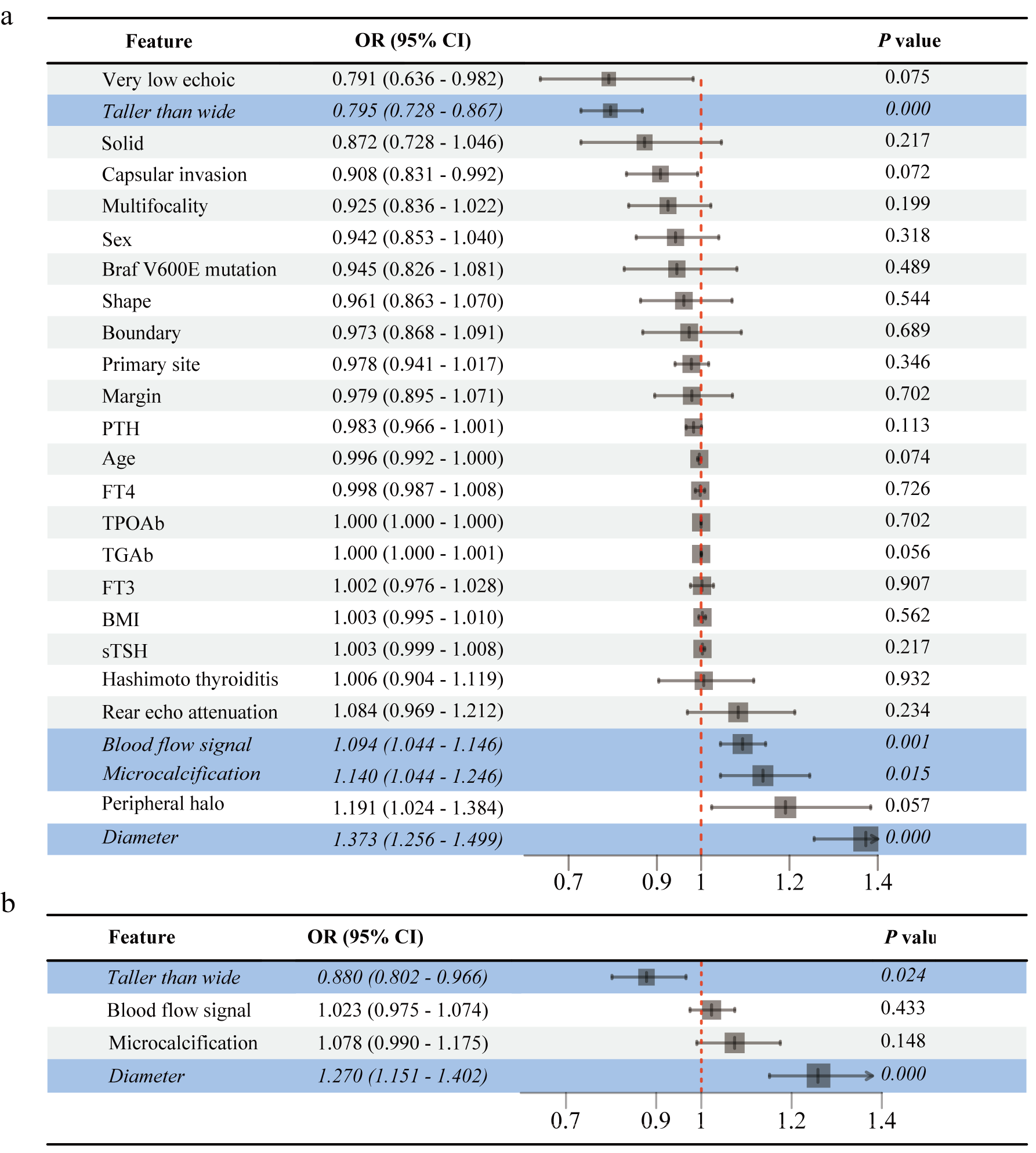

Supplement: S1 Fig — a and b represent the results of univariate and multivariate analysis. (TIF) [file pone.0345937.s001.tif]

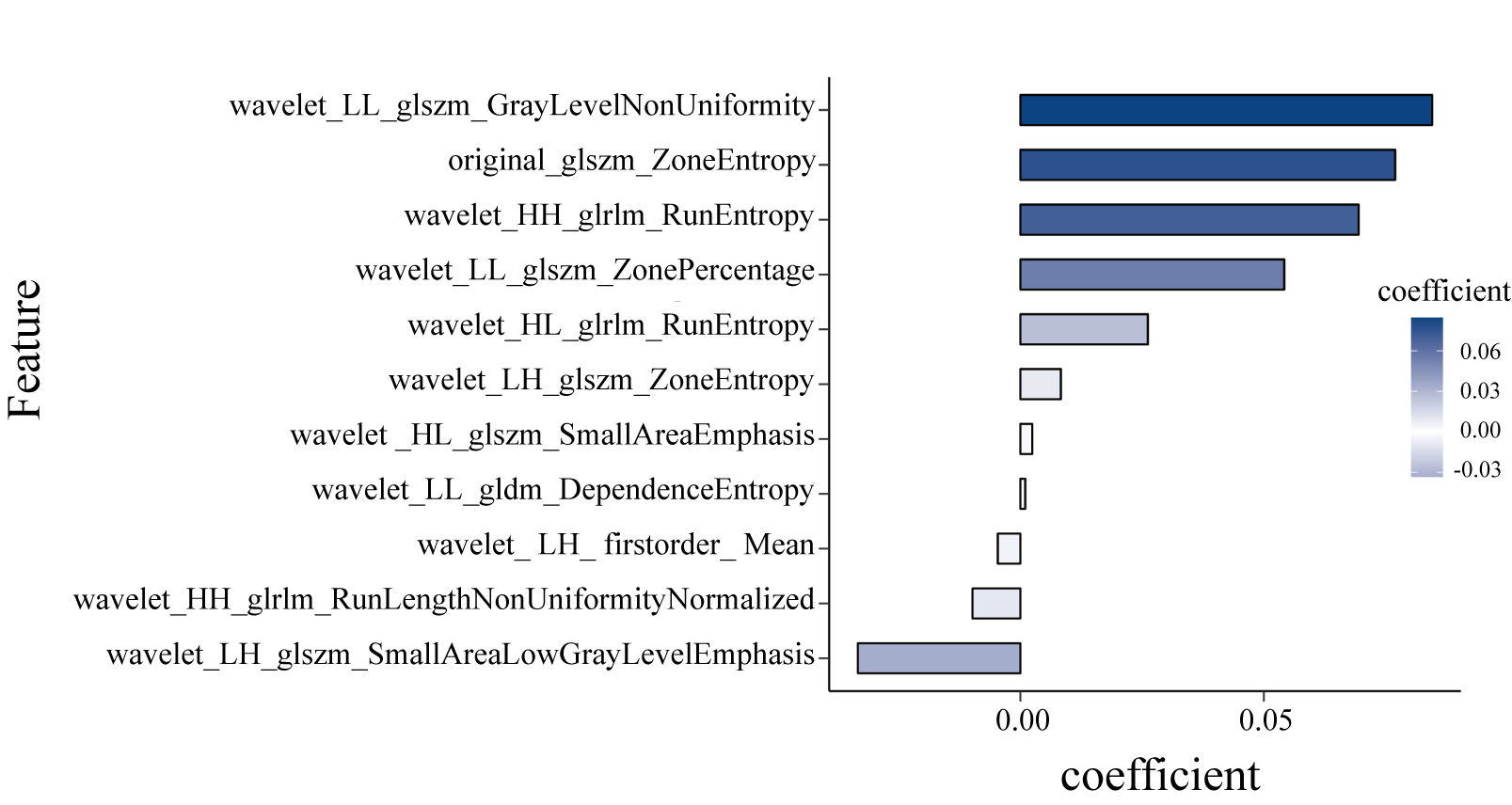

Supplement: S2 Fig — (TIF) [file pone.0345937.s002.tif]

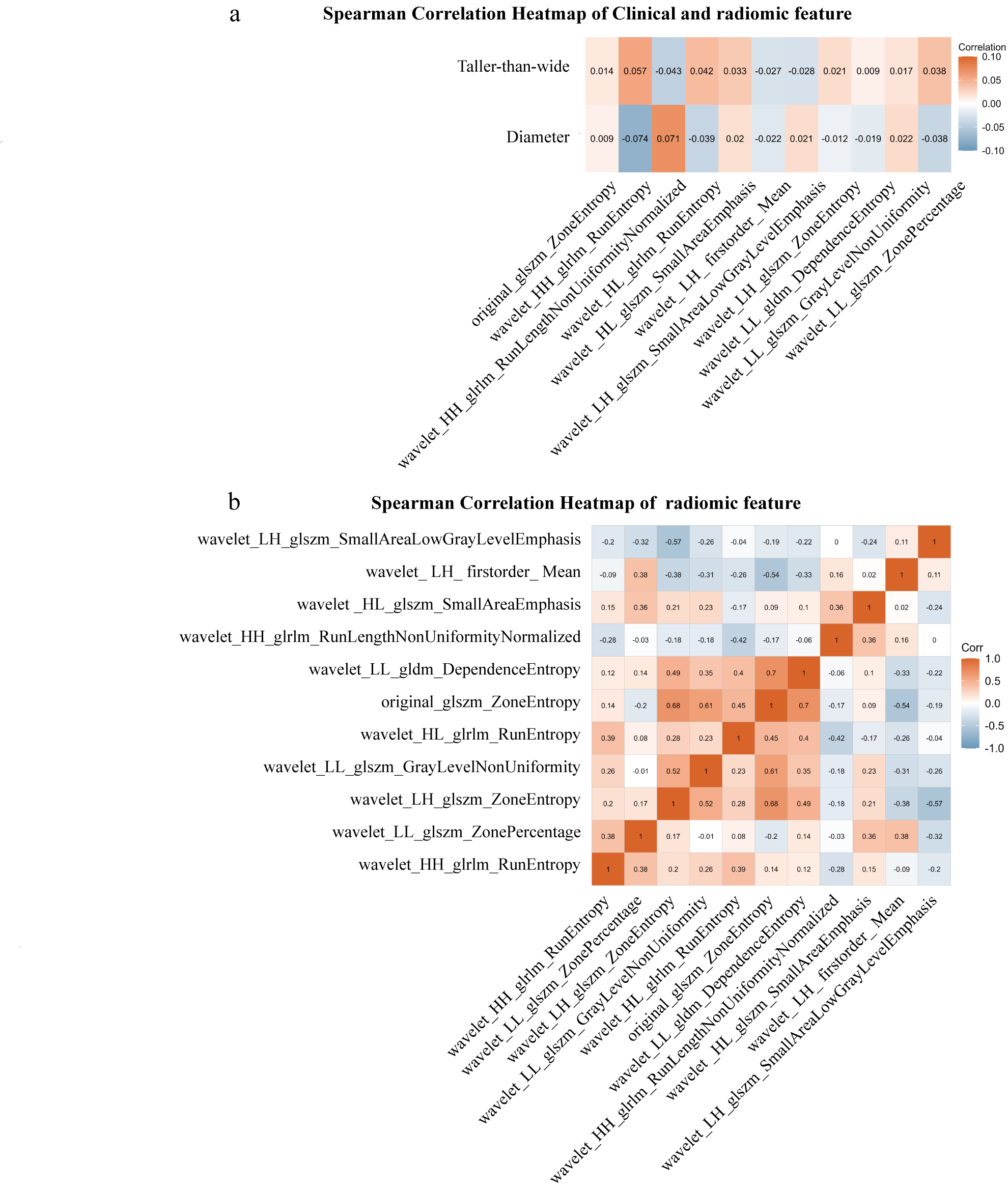

Supplement: S3 Fig — (TIF) [file pone.0345937.s003.tif]
