# Supplementary material for: Utilizing the transformer mechanism to predict cervical lymph node metastasis in patients with papillary thyroid carcinoma
Source: PLoS One. 2026 Apr 3;21(4):e0345937. doi: 10.1371/journal.pone.0345937 (PMC13048401; doi:10.1371/journal.pone.0345937)
Supplement: S1 Table — (DOCX) [file pone.0345937.s004.docx]

| **S1 Table .**Clinicopathological and sonographic characteristics of patients in PTC by cervical lymph node status | | | | |
| --- | --- | --- | --- | --- |
| Features | Training cohort | Internal Validation cohort | External Validation cohort | *P* value |
|  | N=350 | N=88 | N=102 |  |
| Sex,No.(%) |  |  |  | 0.705 |
| Male | 96(27.4%) | 28(31.8%) | 28(27.5%) |  |
| Female | 254(72.6%) | 60(68.2%) | 74(72.5%) |  |
| Age(y) | 46.0(37.0;54.0) | 45.0(37.8;51.0) | 46.0(39.2;53.0) | 0.762 |
| BMI | 23.5(21.3;25.8) | 23.6(21.6;26.2) | 23.8(21.7;26.3) | 0.780 |
| FT3(pmol/L) | 5.24(4.87;5.67) | 5.35(5.03;5.68) | 4.88(4.51;5.24) | <0.001* |
| FT4(pmol/L) | 15.8(14.4;17.5) | 15.9(14.7;17.8) | 15.4(14.2;17.4) | 0.373 |
| sTSH(pmol/L) | 1.63(1.12;2.48) | 1.79(1.23;2.34) | 1.87(1.38;2.86) | 0.052 |
| TPOAb(pmol/L) | 38.7(28.0;66.5) | 37.3(28.0;54.8) | 17.3(14.9;34.3) | <0.001* |
| TGAb(pmol/L) | 4.25(0.80;16.7) | 3.65(0.70;15.0) | 14.0(9.00;29.0) | <0.001* |
| PTH(pmol/L) | 3.55(2.69;4.40) | 3.46(2.61;4.30) | 3.74(2.75;4.54) | 0.423 |
| Primary site(%) |  |  |  | 0.636 |
| Left lobe | 132(37.7%) | 34(38.6%) | 40(39.2%) |  |
| Isthmus | 17(4.86%) | 4(4.55%) | 5(4.90%) |  |
| Right lobe | 146(41.7%) | 38(43.2%) | 40(39.2%) |  |
| Mix | 55(15.7%) | 12(13.6%) | 17(16.7%) |  |
| Multifocality(%) |  |  |  | 0.670 |
| No | 259(74.0%) | 69(78.4%) | 75(73.5%) |  |
| Yes | 91(26.0%) | 19(21.6%) | 27(26.5%) |  |
| Diameter(cm) |  |  |  | 0.986 |
| <1 | 57 (64.8%) | 230 (65.7%) | 67 (65.7%) |  |
| >1 | 31 (35.2%) | 120 (34.3%) | 35 (34.3%) |  |
| Taller-than-wide |  |  |  | 0.855 |
| <1 | 150(42.9%) | 35(39.8%) | 42(41.2%) |  |
| >1 | 200(57.1%) | 53(60.2%) | 60(58.8%) |  |
| Solid(%) |  |  |  | 0.868 |
| No | 22(6.29%) | 5(5.68%) | 5(4.90%) |  |
| Yes | 328(93.7%) | 83(94.3%) | 97(95.1%) |  |
| Very low echoic(%) |  |  |  | 0.631 |
| No | 15(4.29%) | 3(3.41%) | 2(1.96%) |  |
| Yes | 335(95.7%) | 85(96.6%) | 100(98.0%) |  |
| Rear echo attenuation(%) | |  |  | 0.317 |
| No | 282(80.6%) | 77(87.5%) | 84(82.4%) |  |
| Yes | 68(19.4%) | 11(12.5%) | 18(17.6%) |  |
| Margin(%) |  |  |  | 0.236 |
| Clear | 144(41.1%) | 45(51.1%) | 45(44.1%) |  |
| Unclear | 206(58.9%) | 43(48.9%) | 57(55.9%) |  |
| Boundary(%) |  |  |  | 0.989 |
| evenness | 64(18.3%) | 16(18.2%) | 18(17.6%) |  |
| unevenness | 286(81.7%) | 72(81.8%) | 84(82.4%) |  |
| Shape(%) |  |  |  | 0.664 |
| regularity | 76(21.7%) | 19(21.6%) | 18(17.6%) |  |
| irregularity | 274(78.3%) | 69(78.4%) | 84(82.4%) |  |
| Peripheral halo(%) | |  |  | 0.392 |
| No | 317(90.6%) | 78(88.6%) | 96(94.1%) |  |
| Yes | 33(9.43%) | 10(11.4%) | 6(5.88%) |  |
| Microcalcification(%) |  |  |  | 0.566 |
| No | 154(44.0%) | 41(46.6%) | 40(39.2%) |  |
| Yes | 196(56.0%) | 47(53.4%) | 62(60.8%) |  |
| Blood flow signal(%) | |  |  | 0.672 |
| None | 156(44.6%) | 40(45.5%) | 49(48.0%) |  |
| I | 125(35.7%) | 37(42.0%) | 36(35.3%) |  |
| II | 37(10.6%) | 6(6.82%) | 7(6.86%) |  |
| III | 32(9.14%) | 5(5.68%) | 10(9.80%) |  |
| BRAF V600E (%) | |  |  | 0.133 |
| No | 43(12.3%) | 10(11.4%) | 20(19.6%) |  |
| Yes | 307(87.7%) | 78(88.6%) | 82(80.4%) |  |
| Capsular invasion(%) | |  |  | 0.133 |
| No | 186(53.1%) | 56(63.6%) | 51(50.0%) |  |
| Yes | 164(46.9%) | 32(36.4%) | 51(50.0%) |  |
| Hashimoto thyroiditis(%) | |  |  | 0.561 |
| Negative | 272(77.7%) | 73(83.0%) | 80(78.4%) |  |
| Positive | 78(22.3%) | 15(17.0%) | 22(21.6%) |  |
| **Abbreviations**:BMI, body mass index;PTC,papillary thyroid carcinoma;LNM,lymph node dissection;FT3,free triiodothyronine 3;FT4,free triiodothyronine 4;sTSH,sensitive thyroid stimulating hormone;TPOAb,thyroidperoxidase antibodies;TGAb,anti-thyroglobulin antibodies;PTH,Parathyroid Hormone  **NOTE**: Median (range) is reported for continuous and counts (percentage) for categorical variables.Chi-squared or Fisher's exact tests, were used to compare the differences in categorical variables, whereas T-test or Mann-Whitney U test was used to compare the differences in continuous variables, as appropriate.**P*<0.05 | | | | |
