# Supplementary material for: Utilizing the transformer mechanism to predict cervical lymph node metastasis in patients with papillary thyroid carcinoma
Source: PLoS One. 2026 Apr 3;21(4):e0345937. doi: 10.1371/journal.pone.0345937 (PMC13048401; doi:10.1371/journal.pone.0345937)
Supplement: S1 File — (DOCX) [file pone.0345937.s006.docx]

**S1 File.**Parameters and configuration files for radiomic feature extraction

**Parameters and configuration for radiomic feature extraction**

imageType:

Original: {}

Wavelet: {}

featureClass:

shape2D:

firstorder:

glcm:

- 'Autocorrelation'

- 'JointAverage'

- 'ClusterProminence'

- 'ClusterShade'

- 'ClusterTendency'

- 'Contrast'

- 'Correlation'

- 'DifferenceAverage'

- 'DifferenceEntropy'

- 'DifferenceVariance'

- 'JointEnergy'

- 'JointEntropy'

- 'Imc1'

- 'Imc2'

- 'Idm'

- 'Idmn'

- 'Id'

- 'Idn'

- 'InverseVariance'

- 'MaximumProbability'

- 'SumEntropy'

- 'SumSquares'

glrlm:

glszm:

gldm:

ngtdm:

setting:

normalize: true

normalizeScale: 100

interpolator: 'sitkBSpline'

resampledPixelSpacing: [1, 1,0]

padDistance: 10

preCrop: true

force2D: true

force2Ddimension: 0

binWidth: 25

voxelArrayShift: 300
