# Supplementary material for: Utilizing the transformer mechanism to predict cervical lymph node metastasis in patients with papillary thyroid carcinoma
Source: PLoS One. 2026 Apr 3;21(4):e0345937. doi: 10.1371/journal.pone.0345937 (PMC13048401; doi:10.1371/journal.pone.0345937)
Supplement: S2 File — (DOCX) [file pone.0345937.s007.docx]

**S2 File.**Ultrasound features of thyroid nodules and t lymph node

**Features of thyroid nodules** : (1) Location: unilateral lobe, isthmus, and bilateral lobes; (2) Multifocal: tumor number ≥ 2; (3) Largest diameter of the tumor: measuring the maximum diameter of the tumor on a transverse or longitudinal section, divided into <1cm and ≥1cm; (4) Aspect ratio: refers to the positional relationship between the nodule and the skin echo band, with a vertical position indicating an aspect ratio of the nodule's longitudinal and transverse measurements greater than 1 on a transverse or longitudinal section, and a horizontal position indicating an aspect ratio of the nodule's longitudinal and transverse measurements less than 1 on a transverse or longitudinal section; (5) Solid: the nodule is entirely composed of solid tissue and does not contain cystic components; (6) Low/very low echo: the internal echo of the nodule is lower than the echo of the thyroid gland or anterior neck muscle; (7) Posterior echo attenuation: the echo behind the nodule is lower than the echo of the surrounding tissue at the same depth; (8)Indistinct boundaries: the border between the nodule and the surrounding thyroid tissue is unclear; (9) Margin: Smooth margin refers to a nodule with clear, smooth, and intact boundaries, while irregular margin refers to a nodule with small lobulation, spiculation, angulation, etc.; (10) Halo sign: a low echo area surrounding the nodule; (11) Shape: irregular shape indicates that the nodule is mass-like, coarse-lobed, etc.; (12) Microcalcification: point-like strong echo in the nodule with a diameter ≤1mm; (13) Extrathyroidal extension: the nodule invades the thyroid capsule, with the border between it and the extrathyroidal tissues unclear; (14) Internal blood flow signal: level 0, no blood flow signal is found in the tumor mass; level I, a small amount of blood flow, with 1-2 dot-like or thin rod-shaped tumor vessels visible, and the length of the rod-shaped blood flow does not exceed half the diameter of the lesion; level II, moderate blood flow, with 3-4 dot-like vessels or a longer vessel penetrating the lesion, its length can be close to or exceed the radius of the tumor; level III, a large amount of blood flow, with ≥5 dot-like vessels or 2 longer vessels visible; (15) Sonographic changes of the thyroid tissue showing Hashimoto's thyroiditis; (16) Results of BRAF V600E gene test [50]. Figure 1 shows some PTC ultrasound image features.

**The lymph node ultrasound features**: (1) presence of microcalcification within the lymph node; (2) presence of liquefaction or cystic areas within the lymph node; (3) round or near-round shape of lymph node (aspect ratio of lymph node <2); (4) eccentric or absent hilum structure, unclear distinction between cortex and medulla; (5) presence of a hyperechoic mass within the lymph node; (6) rich or relatively rich blood supply, with peripheral or mixed blood flow distribution present. Figure 2 shows some ultrasound features of suspected metastatic lymph nodes in PTC.
